# Dogs motivate obese children for physical activity: key elements of a motivational theory of animal-assisted interventions

**DOI:** 10.3389/fpsyg.2013.00796

**Published:** 2013-10-29

**Authors:** Rainer Wohlfarth, Bettina Mutschler, Andrea Beetz, Friederike Kreuser, Ulrike Korsten-Reck

**Affiliations:** ^1^Department of Public Health & Health Education, University of EducationFreiburg, Germany; ^2^Freiburg Institute of Animal-Assisted TherapyFreiburg, Germany; ^3^Department of Behavioral Biology, University of ViennaVienna, Austria; ^4^Department of Rehabilitative and Preventive Sports Medicine, University of FreiburgFreiburg, Germany

**Keywords:** exercise performance, children, obesity, dog assisted intervention, motivation

## Abstract

**Background:** There is empirical evidence that the presence of a companion animal can have a positive impact on performance. The available evidence can be viewed in terms of differing hypotheses that attempt to explain the mechanisms behind the positive effects. Little attention has been given to motivation as a potential mode of action with regards to human-animal interactions. First we give an overview of evidence that animals might promote motivation. Second we present a study to examine the effect of a therapy dog on exercise performance in children with obesity.

**Methods:** Twelve children, aged 8–12 years old, were randomly assigned to two groups in a crossover design: dog-group and human confederate group. Several types of physical activities via accelerometer and subjective ratings of wellbeing, satisfaction, and motivation were assessed. Data were analyzed using analysis of variance for repeated measures on one factor.

**Results:** The main effect of condition was significant for all performance variables. There was less passive behavior and more physical activity for all performance variables in the presence of the dog than in that of the human confederate. Between dog- and human- condition there was no difference in the subjective rating of motivation, wellbeing, or satisfaction.

**Discussion:** The results demonstrate that the presence of a therapy dog has the potential to increase physical activity in obese children. Task performance as a declarative measure was increased by the presence of the dog in comparison to a human confederate, but self-report measures of motivation, satisfaction or wellbeing did not differ between the two conditions. Therefore, it stands to reason that a dog could trigger implicit motives which enhance motivation for activity. The results of our study indicate the potentially beneficial effect of incorporating dogs into outpatient training for obese children.

## Theoretical background

Animals have played an important role in people's life throughout human history. In spite of the long-lasting presence of companion animals in human life, the idea that interaction with animals may exert a positive effect on human health is rather recent. Epidemiological studies comparing aspects of the health of pet owners and non-pet owners show that adult pet owners are slightly more physically active (Johnson and Meadows, 2010; Peel et al., 2010). In some studies, there was no difference between adult dog-owner and non-dog owner physical activity and most studies show that adult dog-owners did not report reaching recommended levels of physical activity (Bauman et al., 2011). In spite of these mixed results, it is speculated that dog owners are healthier due to walking their dogs (Coleman et al., 2008; Sirard et al., 2011). Dog-owners seem also to recover faster after major illness or surgery (Friedmann and Thomas, 1998; Friedmann et al., 2003; Wisdom et al., 2009; Abate et al., 2011) and make fewer visits to their doctor (Siegel, 1990; Headey and Grabka, 2007). Children with a dog were more likely to achieve the recommended level of weekly physical activity (Christian et al., 2012). Findings are contradictory regarding dog ownership and children's weight status (Timperio et al., 2008; Christian et al., 2012; Westgarth et al., 2012).

The therapeutic relationship in animal-assisted interventions (AAI) differs from living with a companion animal in a social and an intimate relationship in various aspects, e.g., limited period of time for interaction, focus on the client's problems, and needs, goal orientation, roles, and professional codes. Therefore, evidence from studies with pet owners may not be transferable to AAI. Beginning in the 1970s with the inspiring work of the psychotherapists Boris Levinson (1969) and Sam and Elizabeth Corson (Corson et al., 1977) AAI research suggests that many benefits are mainly associated with the supervised interaction with a friendly, calm, trained, and well-groomed animal (Nimer and Lundahl, 2007; Julius et al., 2012). Dogs in particular seem to offer benefits in a variety of settings: therapeutic settings (Prothmann et al., 2006; Beetz et al., 2011), classrooms (Kotrschal and Ortbauer, 2003; Jalongo, 2005), and special needs environments (Anderson and Olson, 2006; Virués-Ortega et al., 2012). And research has demonstrated the considerable positive effects of interacting with animals: physiological (Beetz et al., 2011) such as reduction of stress-related parameters and release of oxytocin (Beetz et al., 2012), emotional and social (Souter and Miller, 2007) such as improvement of mood and positive social interaction, behavioral (Nimer and Lundahl, 2007), physical (Gee et al., 2007) such as better motor skills, and performance-related (Gee et al., 2012) such as better concentration on a task.

Whereas intervention studies provide some evidence of the effect of companion animals on health, many of the mechanisms of AAI remain unclear (Wohlfarth et al., 2013). The available evidence can be viewed in terms of different hypotheses that attempt to explain the mechanisms behind the positive effects (Kruger and Serpell, 2010): anxiety and stress buffering (Julius et al., 2012), social enhancing (McNicholas and Collis, 2000), attachment promoting (Julius et al., 2012; Zilcha-Mano et al., 2011), social support providing, or self-efficacy enhancing (Berget and Ihlebæk, 2011). Little attention has been given to motivation as a potential mode of action (Olbrich, 2009a,b) with regard to human-animal interactions. However, we are proposing that animals might promote motivation and will therefore explain this framework of our study in more detail.

In recent years theories of motivation have mainly been based on the distinction between implicit and explicit motive systems (Schultheiss et al., 2012). In particular, the possibility that implicit and explicit motive systems might conflict with each other is a central proposition of the dual-system approach to motivation. Research suggests that congruence of implicit and explicit motive systems is associated with low intrapersonal conflict, high intrinsic motivation, and successful performance—the preconditions for happiness, well-being, and health (Schultheiss and Brunstein, 2010). Implicit motives have been conceptualized as associative networks connecting situational cues with basic affective reactions and implicit behavioral tendencies (Schultheiss et al., 2012). We suggest that in AAI fulfillment of any implicit motive is associated with task enjoyment and intrinsic motivation—not just fulfillment of certain “fundamental” implicit motives or needs (Schultheiss and Brunstein, 2010).

In contrast to explicit motives, implicit motives are built on associations with innately triggered affective, mainly non-verbal experiences, called “natural incentives” (McClelland et al., 1989). In general, it is proposed that implicit motives are subconsciously aroused and mainly respond to non-verbal stimuli, while explicit motives are consciously aroused and respond mainly to verbal stimuli (Stanton et al., 2010). Schultheiss et al. (2008) have also suggested that the pursuit of goals that are supported by a person's implicit motives represents a hot and affectively engaging mode of goal-striving, whereas the pursuit of goals that are not supported by a person's implicit motives represents a cold, affectively neutral mode. So, implicit motives represent capacities for specific affective experiences and orient, select, and energize behavior (Schultheiss and Brunstein, 2010). Research shows that implicit motives influence non-declarative measures such as task performance, attention orienting, and physiological changes, but not declarative measures such as choices, attitudes, and judgments, and the reverse is true for explicit motives (Schultheiss and Köllner, 2013). Therefore, implicit motives cannot be measured via self-reports but indirectly via non-declarative measures employing performance criteria.

We propose that implicit motives may be an important mode of action in AAI.

1. Implicit motives are mainly processed by the experiential system (Schultheiss, 2001) and the experiential system processes a reality that is similar to an animal's world, because it is comprised of the sights and sounds, the smells and sensations that impinge on our senses every second. Such experiential stimuli include, for example, the sight of a friendly dog, the waving of a tail or the fur touching one's skin. Their meaning for the organism is coded by positive emotional-motivational states, such as curiosity, affection, or joy.
2. Implicit motives are non-conscious. Implicit motives respond to incentives which are perceived and represented non-verbally. Facial expressions of emotions are particularly salient non-verbal cues for implicit motives (Stanton et al., 2010). So, the body language of animals may interact with individuals' implicit motives. A friendly tail-waving dog may trigger the affiliation motive. A dog which seems to be listening to a story read by a child may trigger the achievement motive.
3. Animals can elicit implicit motives because all mammalian species share fundamental evolutionarily preserved motivational systems that propel them toward the formation of attachments and ensure safety and protection (Hall et al., 2010, p. 279). It is proposed that implicit motives are closely tied to regions of the “emotional brain” (LeDoux, 1996). Human-animal interaction research has documented that interaction with animals (by playing or petting) increases or decreases several neurohumoral agents: decrease of the cortisol (Beetz et al., 2012) and increase of serotonin and dopamine (Odendaal and Meintjes, 2003). These changes in hormone levels when interacting with an animal may be a link to our motivation system.

The effects found in AAI show a few principal similarities with mediators of children's physical activity. Reviews of potential mediators show that physical activity is influenced by multiple factors: self-efficacy (Van der Horst et al., 2007; Brown et al., 2013), parental support or feedback (Weiss, 2004; Kahn et al., 2002; Allender et al., 2006; Rees et al., 2006; Van der Horst et al., 2007; Brown et al., 2013); parental physical activities (Sallis et al., 2000; Rees et al., 2006; Van der Horst et al., 2007), self-competencies (Weiss, 2004; Allender et al., 2006; Rees et al., 2006; Brown et al., 2013), enjoyment (Weiss, 2004; Allender et al., 2006; Brown et al., 2013). Additionally, in a recent review knowledge about physical activities seems to be a decisive factor (Brown et al., 2013). In summary interventions enhancing physical activity in children should include optimal challenges, a mastery motivational climate, enjoyment, achievable goals, and foster feelings of competence (Weiss, 2004).

Based on motivation theory (Schultheiss, 2001), we claim that animals influence mainly implicit motives. In performance and learning situations AAI may enhance implicit motives and improve the implicit-explicit motive-goal congruence (Olbrich, 2009a,b). Making exercises for and with a dog would thus lead to a higher level of implicit motivation, which would predict higher performance. The higher implicit motives could be measured indirectly via a better performance in the presence of a dog. However, no effects on self-report measures are to be expected.

## Aims

The purpose of this study was to examine the effect of a therapy dog on exercise performance in children with obesity. Specifically, we investigated whether a group of children aged 8–12 years would profit more from the presence of a dog in contrast to the presence of a friendly person while performing a variety of movement games. In the presence of a dog, the children should gain more pleasure from these activities and perform better, since we propose that these stimuli and events are affectively hot and therefore implicit motivation is strong. The dog may serve as the catalyst for the activation of implicit motives, which support movement performance. There should be no effect of a therapy dog on self-report measures of motivation and satisfaction.

## Methods

### Sample

Twelve children aged 8–12 years, six males, and six females, who participated in the Freiburg Intervention Trial for Obese Children (FITOC) were included in the study. FITOC is an interdisciplinary, outpatient program for obese children, consisting of regular physical exercise and comprehensive dietary and behavioral education. The mean age of the children was 9.67 years (*SD* = 1.93). All children had a BMI between the 90th and 97th percentile. Mean BDI-SDS was 2.30 (*SD* = 0.30).

All children participated on a voluntary basis after approval in verbal form. Written consent was obtained from the parents. The study was conducted in accordance with the ethical principles of the Declaration of Helsinki.

### Design

#### Condition

There were two experimental conditions which were determined by one of the following co-performers of the movement tasks:

1. Real dog: A white Hunting dog (female, neutered, 2 years of age, German Kennel Club's Canine Good Citizen, certified as Therapy Dog according to the standards of the European Society for Animal Assisted Therapy). The children had several opportunities to interact with the dog prior to the experiment.
2. Human confederate: The human confederates were a 40-years-old woman and a 23-years-old woman. Both had also made a number of visits to the children prior to the experiment so that their presence was not a novelty to the children. These persons were present during both conditions. The confederates tried to enhance motivation via verbal support. They assigned and described the goals to the children verbally and encouraged them to try their best.

#### Procedure

All 12 children underwent both conditions. The 12 children were randomly assigned to wear the accelerometer in two groups in a crossover design. Three boys and three girls performed first in the dog condition and at the next session in the human condition (Order 1). Three boys and three girls first had the human condition and then the dog condition (Order 2).

In both orders, similar exercise programs were feasible. The exercise programs consisted of retrieving, agility, speed play, and burning ball. During the sessions the dog was only active twice for 10 min. The rest of the time the children did things for the dog.

### Examples of exercises

“Agility with dog”: Part 1: The dog runs an agility course. The children were instructed to try to be faster than the dog. Part 2: Children take food with a spoon, or water with a sponge, through the agility course.
“Agility with human confederate”: Part 1: The human confederate runs an agility course. The children try to be faster than the confederate. Part 2: Children take small figures with a spoon, or water with a sponge, through the agility course.

For both conditions there were two courses with six children. The agility exercises lasted about 20 min. Each child completed about six rounds.

“Dog retrieving”: Like in baseball, children aim to make as many rounds as possible while the dog is retrieving a dummy, thrown by one of the children. Children who did not reach a base before the dog returned the dummy lost a point.
“Human confederate retrieving”: Similar to “retrieving with a dog” but a human confederate retrieves a ball.

For both conditions all 12 children played the motion game together for about 20 min per session.

### Experimenter

The procedures were supervised and conducted by the same experimenter who was familiar with the children and the human confederates prior to the study. The experimenter also observed and documented the human-dog interaction.

### Dog handler

The dog handler was a 32-years-old woman who was present in both conditions. She interacted with the children as little as possible.

### Instruments

#### Accelerometer

Physical activity as a non-declarative measure of movement performance was assessed using an accelerometry-based motion sensor (AiperMotion 440T, Aipermon GmbH, Germany). The system uses 3D acceleration sensors and analyzes data with a disclosed online algorithm. The algorithm calculated the “active” acceleration rates into four activity levels which were based on an unpublished pilot study. In the pilot study we observed the children while performing different activities. We evaluated the different activities by direct observation measuring exact acceleration rates after every activity to precisely define the intensity classes. The four classes were divided into different degrees of acceleration (acceleration rates). The accelerometer recorded the activity from the moment it was turned on. Activity distribution was calculated for each child in the assessed time periods. There was a high correlation between time periods of the observed and measured intensity classes. Also other studies show that accelerometers are reliable in measuring physical performance (Trost, 2007; Jehn et al., 2009; Ekelund et al., 2011). The children were fitted with the accelerometer by a trained research staff member. Subjects were requested to wear the accelerometer on a belt at the hip during the sessions.

#### Questionnaire about the activities and personal data

For the assessment of wellbeing, motivation, and satisfaction with the activities we used a 5-point Likert scale for each item with smileys as a declarative measure of self-report. After each session each child completed a questionnaire about the activities.

### Statistical analysis

Data were analyzed with SPSS 21.00. To determine the effect of the factors “condition” and “order” on the exercise performance parameters, we conducted a Two-Way Mixed-Model Factorial ANOVA. Statistical significance was determined based on an alpha level of 0.05. No adjustment for multiple comparison testing was performed because of the explorative character of the study. Partial η^2^ values were used as indicators of the effect size of each factor. According to definitions of effect size, small, medium, and large effects for η^2^ are considered to be 0.10, 0.25, and 0.40 for “small,” “medium,” and “large.”

## Results

The performance data assessed via the accelerometer were subjected to a Two-Way Mixed-Model ANOVA (see Table 1).

**Table 1:** **Performance data assessed via accelerometer in minutes; Two-Way Mixed-Model ANOVA for condition and order**.

	**Human**		**Dog**		**Statistical parameters**
	***M***	***SD***	***M***	***SD***	
Overall time	55.63	4.11	57.41	3.58	*F* = 0.91, *p* < 0.360, η^2^ = 0.084
Passive	22.81	2.81	14.21	2.41	*F* = 121.36, *p* < 0.000, η^2^ = 0.924
Motion without steps	14.54	2.65	17.20	3.98	*F* = 13.091, *p* < 0.005, η^2^ = 0.567
Slow walking	12.08	2.53	13.78	2.99	*F* = 13.548, *p* < 0.004, η^2^ = 0.575
Fast walking	5.97	2.77	11.51	4.10	*F* = 40.636, *p* < 0.000, η^2^ = 0.803
Sportive	0.22	0.29	0.71	0.64	*F* = 12.445, *p* < 0.005, η^2^ = 0.554

The overall time of wearing the accelerometer did not differ between order (*F* = 0.919, *p* < 0.360, η^2^ = 0.084) or conditions (*F* = 2.611, *p* < 0.137, η^2^ = 0.207). Effect size was only small.

The main effect of condition was significant for all performance variables. There was less passive behavior (e.g., sitting, standing or lying) in the presence of the dog in comparison to the human confederate (*F* = 121.36, *p* < 0.00, η^2^ = 0.924). The effect size for this analysis was found to exceed convention for a large effect. There was no statistically significant effect of order on all performance variables.

The physical activity was higher for all five performance variables in the dog condition: Children showed more active behavior, which means physical activity without making steps (e.g., sitting and moving the upper body) over a longer period of time in the dog condition (*F* = 13.091, *p* < 0.005, η^2^ = 0.567). Also, children spent more time with slow walking in the dog condition than in the human condition (*F* = 13.548; *p* < 0.004, η^2^ = 0.575). For slow walking there was a slight, but statistically insignificant, order effect. Differences in Order 2 were more pronounced (*F* = 3.549, *p* < 0.089, η^2^ = 0.262). In spite of a statistically insignificant effect, the effect sizes were found to exceed convention for a medium effect. This indicates that an order effect may have occurred in a larger sample. Children having the human first and then the dog condition spent more time slow walking than children having the dog first and then the human condition.

The most remarkable difference between the dog and human condition however was that the children walked faster over a longer period of time in the presence of the dog (*F* = 40.636; *p* < 0.000, η^2^ = 0.803). Also, sportive activity was higher when the dog was present (*F* = 12.445, *p* < 0.005, η^2^ = 0.554).

There was no difference between dog and human condition in the subjective rating of motivation (*F* = 0.918, *p* < 0.360, η^2^ = 0.084) or wellbeing (*F* = 2.813, *p* < 0.124, η^2^ = 0.220). There was a small difference for satisfaction which, however, did not reach statistical significance (*F* = 4.310, *p* < 0.065, η^2^ = 0.301). The size of the condition effect for wellbeing and satisfaction can be classified as medium. There was no order effect for all three subjective ratings.

## Discussion

The regulation of activities can be amotivated, extrinsically motivated or intrinsically motivated. Amotivation is a state characterized by a lack of intention to engage in the activity. Extrinsic motivation implies that a person engages in the behavior to satisfy an external requirement, to avoid negative feelings or to enhance one's ego (Ryan and Deci, 2000). Intrinsic motivation represents the most powerful type of motivation and refers to engaging in the activity for its own sake. An intrinsically motivated person considers the activity inherently enjoyable, interesting, and challenging (Deci and Ryan, 2012). There is evidence that a prerequisite for intrinsic motivation is that the behavior is congruent with actual affective preferences stemming from aroused implicit motives (McClelland, 1985). We propose that intrinsic motivation does not depend on support from cognitive preferences. Many children attending AAI, for example, do not feed an animal just to satisfy the hunger of the animal, but rather feed the animal for the intrinsic joy of feeding. Moreover, it seems likely that intrinsic motivation from aroused implicit motives is accompanied by more profound experiences of meaning and purposefulness only if aroused implicit and activated explicit motives are congruent.

The data presented above demonstrate that the presence of a therapy dog has the potential to increase physical activity in obese children aged 8–12 years. Children profited more from the presence of a dog than from a friendly person when participating in a variety of movement games. Therefore, it stands to reason that a dog could trigger implicit motives which enhance motivation for activity. Based on motivation research and our findings, we propose that in the presence of a dog children gain more pleasure from the activity, because a dog serves as an affectively hot stimulus and a natural incentive, therefore the implicit motivation for activity was strong. The dog subconsciously addresses implicit motives which lead to affective preferences and implicit behavioral impulses. Therefore, the dog may have served as catalyst and accelerator for the activation of implicit motives, which enhances intrinsic motivation and further movement performance. Stanton et al. (2010) showed that body language expressions of emotion are salient non-verbal cues for implicit motives. A dog expresses emotion non-verbally and communicates mainly with body language and therefore the cues sent by the dogs are in a pre-existing experiential format and can therefore serve as a motivational incentive for implicit motives. Hence, they are able to promote physical activity.

The results of this study are particularly noteworthy because the dogs were only active for 20 min within a 90-min session. Therefore, therapy dogs seem to be a powerful motivational force for more activity.

Moreover the findings demonstrate that a human confederate who assigned and described a goal verbally to the children was less successful in promoting physical activity. It should be noticed that intrinsic motivation does not include perceived abilities. Low perceived abilities do not preclude intrinsic motivation, but low perceived abilities preclude flow experience (Csikszentmihalyi and Csikszentmihalyi, 1988). Flow describes a specific case of intrinsic motivation characterized by undivided attention to the task, impaired sense of time, and absence of intrapersonal conflict and self-referential or other distracting thoughts (Csikszentmihalyi and Csikszentmihalyi, 1988). Low perceived abilities counteract the experience of flow. Hence, congruent implicit and explicit motives, which are the sufficient condition for intrinsic motivation, must combine with perceived abilities to allow for a flow experience. Here, dogs may serve as models for new abilities, because children realize that they could do the same as the dog. We propose that dogs enhance implicit motives and further intensify the congruence of implicit and explicit motives. The implicit-explicit motive congruence and perceived abilities are associated with flow experiences which result in higher physical activity.

As in previous research, results of our study suggest that implicit motives are associated only with non-declarative measures of motivation, such as hormone changes, cardiovascular responses, response speed on performance tasks, and intuition-guided behavior (Schultheiss and Brunstein, 2010). In our study, task performance as a declarative measure was increased by the presence of the dog compared to a human confederate, but self-report measures of motivation, satisfaction, or wellbeing were statistically insignificant between the two conditions. Due to the medium effect sizes between the conditions for wellbeing and satisfaction the above conclusion must be drawn with care. The statistically insignificance might due to the small sample size. It seems that especially non-declarative measures can be positively influenced by AAI (e.g., Odendaal and Meintjes, 2003; Miller et al., 2009; Gee et al., 2010; Beetz et al., 2011). Thus, the implicit system, which is geared toward processing non-verbal information and generates automatic, incentive-driven behavior aimed at maximizing pleasure (Schultheiss and Brunstein, 2010), may be directly affected by animals.

## Conclusion

Research suggests that AAI are more effective in children, the elderly, and persons with impairments or diseases. This can be explained by the differential effect of animals on the two different motivation systems. Animals may address the experiential system of motivation more than the verbal-symbolic system. Children are able to differentiate and emotionally respond to emotional body language expressions long before they are able to verbally label those expressions. And similarly elderly people, especially when suffering from dementia, respond more to experiential-emotional cues than to verbal ones. Motivation theory can make a significant contribution to understanding the mechanisms of AAI.

The results of this study are also noteworthy because statistically significant effects were found with such a small sample size (*n* = 12). Generally, effects of this nature are more likely to be of practical importance than the result of power and effect size being artificially increased by a large sample size. Larger sample sizes reduce the standard deviation of the comparison distribution, thus functionally increasing both power and effect size. On the other hand, one could argue that because of the small sample size this particular sample may not be representative of the larger population of obese children in general.

Future research should test whether such a dissociation between declarative and non-declarative measures of motivation may also be found in adults and be directed at developing valid, reliable, and economical instruments for exploring the motivational effects of AAI—notably, implicit and explicit motives and intrinsic and extrinsic motivation.

However, the results of our study indicate the potentially beneficial effect of incorporating dogs into outpatient training for obese children. Childhood obesity is a major public health concern, with a rapidly rising prevalence. Childhood obesity is an important predictor of adult obesity with potential cardiovascular risk factors (e.g., Whitaker et al., 1997). Overall, the socioeconomic costs of childhood obesity are enormous (Wang and Dietz, 2002).

There are a number of programs for the prevention and therapy of obesity which combine regular physical exercise with theoretical and practical information about nutrition, as well as background information about psychological and physiological problems. The major problem with these programs is that overweight children prefer physically inactive activities like playing computer games or watching television (Maffeis, 2000; Kreuser et al., 2013). Therefore, physical activities should first and foremost be fun and have a high motivation factor. Our results suggest that incorporating dogs in programs for overweight children may help to motivate children to undertake physical exercise and promote sustainable lifestyle changes.

### Conflict of interest statement

The authors declare that the research was conducted in the absence of any commercial or financial relationships that could be construed as a potential conflict of interest.
